# Teleradiologic network for central management and analysis of MR images: a three-years experience of the competence network for congenital heart disease

**DOI:** 10.1186/1532-429X-11-S1-O49

**Published:** 2009-01-28

**Authors:** Samir Sarikouch, Philipp Beerbaum, Stefan Mueller, Ulrich Sax, Titus Kuehne

**Affiliations:** 1 Department for Heart-, Thoracic-, Transplantation- and Vascular Surgery, Hannover Medical School, Germany; 2 Division of Imaging Sciences, King's College, Guy's & St Thomas' Hospital, London, UK; 3 Department for Medical Informatics, University of Goettingen, Goettingen, Germany; 4 Unit of Cardiovascular Imaging – Congenital Heart Diseases, German Heart Center, Berlin, Germany

**Keywords:** Congenital Heart Disease, Consumption Cost, Competence Network, Invaluable Source, Data Transmission Speed

## Background

Analysis of cardiovascular MRI requires increasingly high standards and produces costs for specially trained personal and technical equipment. At the same time clinical decision making relies, particulary in congenital heart diseases (CHD), more and more on MRI derived data. In the German Competence Network for CHD, a science-association was founded in which MRI data were acquired in peripheral institutes and than send by teleradiologic means to a core-labaratory for central data analysis and archiving. The first three years of operation of this network were evaluated regarding its technical course of activity, accuracy of MRI data analysis and costs.

## Methods

16 cardiovascular institutes form part of the network. MR images are acquired in these institutes (Philips, Siemens, GE scanners) using a standardized protocol. Images are send by teleradiologic means (pseudonomyzed via internet) to a central core-laboratory. There, images are analysed for parameters of cardiac function using custom-made software. Variability of the measured parameters is continuously determined (quality assessment). Thereafter, analysed data and original MR images are archived and are accessible to the participating institutes by a remote-data-entry-system (RDE). Economic evaluation of the network was done by cost-analysis and ex post-contemplation under consideration of investments, labor- and consumption-costs.

## Results

Data transmission speed was approximately 300 kbit/s upstream. MR images arrive at the core-laboratory as pseudonymized DICOM-data. After the pilot phase, no errors in image transmission (e.g. incomplete data sets) were observed. Therefore, all MRI scans that were acquired using standardized protocols could be analysed in the core-labaratory, so far more than 1000 scans. Interobserver variability of quantitative function parameters was significantly lower when analysis was done in the core-labaratory, compared to individual analysis in the peripheral institutes. MR images were ubiquitously accessible in the RDE-system and via the internet at any time. Ex-post evaluation showed an expenditure of 155 EUR per MRI data set when 552 sets per year are enrolled under scientific (non economic) conditions to the core-labaratory.

## Conclusion

A teleradiologic network was successfully established in a science-association for congenital heart disease and optimized for primary health care and scientific purposes. The first three-year experiences showed that this network/core-lab is attractive by reducing labor-, investment- and consumption costs and at the same time improves quality of data. Finally, the data archives of the core-labs are a very large and invaluable source of data for current and future scientific studies.

